# High expression of prolyl 4-hydroxylase subunit alpha-2 in lung adenocarcinoma indicates poor prognosis

**DOI:** 10.1016/j.clinsp.2022.100123

**Published:** 2022-11-17

**Authors:** Xiao-Hong Lu, Die Sang, Yu-Rong Zhang, Qing Yuan

**Affiliations:** a Department of Medical Oncology, Beijing Chao yang District San huan Cancer Hospital, Beijing, China; b Department of Neurosurgery, Center for Cancer Precision Medicine, National Cancer Center/National Clinical Research Center for Cancer/Cancer Hospital, Chinese Academy of Medical Sciences and Peking Union Medical College, Beijing, China

**Keywords:** Lung adenocarcinoma, Brain metastases, P4HA2, Pprognosis, Biomarker

## Abstract

**Objective:**

To analyze the Prolyl 4-Hydroxylase subunit Alpha-2 (P4HA2) expression in Lung Adenocarcinoma (LAUD).

**Methods:**

The authors assessed P4HA2 expression in the LUAD tumor ecosystem using single-cell analysis. The authors analyzed the relationship between P4HA2 expression and clinical features in LUAD and Brain Metastasis (BM) cases. The authors assessed the biological functions of P4HA2 using The Cancer Genome Atlas-LUAD dataset.

**Results:**

P4HA2 was more highly expressed in fibroblasts than in epithelial cells in normal lung and lung adenocarcinoma tissues (p < 0.001). P4HA2 was more highly expressed in malignant epithelial cells than in fibroblasts in the BM tissue (p = 0.002). P4HA2 expression was significantly higher in female cases than in male cases (p = 0.049) and was related to lymph node metastasis (p = 0.019) and a higher TNM stage (p = 0.020). High P4HA2 expression indicated a poor prognosis and served as an independent prognostic risk factor in lung cancer. P4HA2 was mainly enriched in the extracellular matrix organization, NADH regeneration, and canonical glycolysis. P4HA2 expression was negatively correlated with naive B cells, T-cells, CD8, and activated natural killer cells, but positively correlated with CD4 memory-activated T cells, regulatory T-cells, resting dendritic cells, and dendritic cell activation. P4HA2 messenger RNA expression was correlated with programmed death-ligand 1 and cytotoxic T-lymphocyte-associated protein 4.

**Conclusion:**

P4HA2 is highly expressed in LUAD tumor cells, especially for the BM subtype, and is a valuable prognostic indicator of LUAD. It may be involved in a biological activity of distant metastasis of LUAD tumor cells and serve as a potential treatment target.

## Highlights

• P4HA2 is highly expressed in LUAD tumor cells, especially for the BM subtype.
• P4HA2 is a valuable prognostic indicator for LUAD.
• P4HA2 is related to biological processes promoting metastasis.

## Introduction

Lung cancer accounts for over 20% of all cancer-related deaths.^1^ Lung Adenocarcinoma (LUAD) represents more than half of all lung cancer cases. Over the last decade, treatment of LUAD, including surgical resection, chemotherapy, and radiotherapy, especially treatment regimens that target Epidermal Growth Factor Receptor (EGFR) and Anaplastic Lymphoma Kinase (ALK), has achieved substantial progress.^2^^,^^3^ However, the 5-year Overall Survival (OS) rate of LAUD is < 20%.^1^ Distant metastasis, especially in the brain, has frequent occurrences and, in most cases, is responsible for unfavorable outcomes.^4^ Thus, exploring the molecular mechanisms of distant metastasis of LUAD tumor cells may help to find therapeutic targets and treatment options and thus improve the prognosis of the disease.^5^^,^^6^

Prolyl 4-Hydroxylase subunit Alpha-2 (P4HA2) is a component of the prolyl 4-hydroxylase family, a key enzyme in collagen synthesis, and is composed of two identical alpha subunits and two beta subunits.^7^ P4HA2 induces remodeling of the Extracellular Matrix (ECM) under hypoxic conditions.^8^ Daniela et al. demonstrated that P4HA2 accumulated in lung cancer cells harbored RASSF1A promoter methylation and participated in the acceleration of collagen deposition and metastatic dissemination in vivo.^9^ Studies have confirmed that P4HA2 participates in the metastasis of oral squamous carcinoma, breast cancer, and prostate cancer.^10^, ^11^, ^12^, ^13^ In brain tumors, P4HA2 promotes cell proliferation and migration in glioblastoma through the PI3K/AKT pathway.^14^ Recently, variation in P4HA2 expression has been observed in multiple cancers, e.g., oral cavity squamous cell carcinoma and cervical cancer, and has been correlated with the prognosis of patients.^12^^,^^15^ However, the expression and clinical significance of P4HA2 in LUAD remains unclear. Therefore, in this study, The authors analyzed the expression of P4HA2 in LAUD and investigated the biological functions of P4HA2 in LUAD.

## Methods

### Data mining from public databases

Gene expression data of RNA-seq and corresponding clinical information from The Cancer Genome Atlas (TCGA) were downloaded with the R package “TCGAbiolinks”. Single-cell RNA sequencing data from a previous study^16^ were explored online at http://ureca-singlecell.kr.

### Patients and TMA construction

Patients with lung adenocarcinoma brain metastases underwent surgical resection in the Department of Neurosurgery at the National Cancer Center/Cancer Hospital of the Chinese Academy of Medical Sciences between November 2012 and June 2019 and were enrolled in this study. Clinical parameters including baseline and pathological information were collected. This research was approved by the Ethics Committee of the Cancer Hospital, Chinese Academy of Medical Sciences (NCC2021C-516).

TMAs were constructed as previously described.^17^ One section from the tissue array block was stained with H&E to confirm that the punches contained tumor tissue. Two experienced investigators reviewed the slides independently to determine and marked representative areas of tumor tissue.

### Bioinformatics analysis

Spearman correlation analysis was performed to identify genes significantly related to P4HA2 in LUAD (|r| > 0.2 and p < 0.05). Gene Ontology (GO) functional analysis and Kyoto Encyclopedia of Genes and Genomes (KEGG) of these genes were performed by the cluster Profiler package.

### In situ detection

Anti-P4HA2 antibody (1:100, 13759-1-AP, Proteintech) was used in the IHC assay. The slides were scanned using a NanoZoomer (Hamamatsu, Japan) high-resolution scanner, and the results were scored blindly with no information on clinical data. P4HA2 expression levels were determined on the basis of staining intensity and the percentage of positive cells. Staining intensity was rated as 0 (negative), 1 (weakly positive), 2 (moderately positive), and 3 (strongly positive). The percentage of positive cells was graded as 0 (0%), 1 (1%‒20%), 2 (21%‒50%), and 3 (51%‒100%). The staining score for P4HA2 was calculated by multiplying the intensity by the positive percentage grade.

### Immune infiltration analysis

The relationship between the expression of P4HA2 mRNA and Tumor-Infiltrating Immune Cells (TIICs) was analyzed. The transcript expression data of TCGA-LUAD were loaded in R and calculations were performed using the “CIBERSORT” package. LUAD individuals were eligible to be entered into the subsequent analysis after quality filtering (p-value < 0.05). After that, the authors compared the difference in containment for all the TIICs between the two groups using the Mann-Whitney *U* test.

### Survival analysis

Survival and survminer packages were loaded in R. Surv_cutpoint function were used to determine the optimal cut-off point of P4HA2 expression in TCGA-LUAD and LUAD-BM.

### Statistical analysis

Significant differences between the two groups were determined by the Mann-Whitney *U* test. The χ2 test was used to assess the relationship between molecular alterations and clinicopathological parameters. Overall Survival (OS) curves were plotted according to the Kaplan-Meier method, with the log-rank test applied for comparison. Multiple Cox regression analysis was used to predict independent prognostic factors. All statistical analyses were performed using both IBM SPSS Statistics 21.0 and GraphPad Prism 5.0; p < 0.05 was considered statistically significant. All tests were two-sided.

## Results

### Study population

In total, 513 LUAD cases from TCGA were collected for further analysis. 113 LUAD-BM cases from the present institution met the indications for surgical resection in BM, including single BMs and multiple resectable BMs, especially with raised intracranial pressure or neurological impairment. After excluding patients without information on living status, 504 TCGA-LUAD cases and 94 LUAD-BM cases were included in the survival analysis. Patients’ baseline information is displayed in Table 1.

**Table 1:** Baseline information for patients enrolled in the analysis.

Variables	TCGA-LUAD	LUAD-BM
Gender		
Male	237	60
Female	276	53
Age (years)		
≤ 60	157	51
> 60	354	62
NA	2	0
T		
T1 (contains T1a and T1b)	168	0
T2 (contains T2a and T2b)	276	0
T3	47	0
T4	19	0
NA	3	113
N		
N0	331	0
N1	95	0
N2	74	0
N3	2	0
NA	11	113
M		
M0	344	0
M1	25	113
Mx	140	0
NA	4	0
Stage		
I	274	0
II	121	0
III	84	0
IV	26	113
NA	8	0
EGFR mutation		
Mutation	80	22
Wild type	193	19
NA	240	72
ALK mutation		
Mutation	34	0
Wild type	209	0
NA	270	113
Living status		
Live	321	50
Dead	183	44
NA	9	19
Total	513	113

### P4HA2 expression in the LUAD tumor ecosystem

**Fig. 1:**
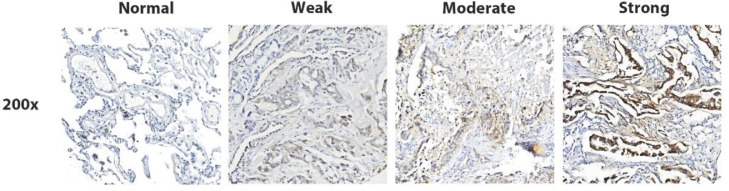
P4HA2 expression in the tissue ecosystem. (A, C, E) tSNE visualization shows P4HA2 expression in different clusters of normal lungs, LUAD, and LUAD brain metastasis tissues, respectively. (B, D, F) Difference in the P4HA2 expression between epithelial cells and fibroblasts of normal lung, LUAD, and LUAD brain metastasis tissues, respectively. P4HA2, Prolyl 4-Hydroxylase subunit Alpha-2; tSNE, t-distributed Stochastic Neighbor Embedding; LUAD, Lung Adenocarcinoma.

Single-cell analysis was performed to explore the difference in P4HA2 expression between heterogeneous cell types in normal lung tissue, LAUD tissue, and BM tissue. The authors found that P4HA2 was more highly expressed in fibroblasts (decorin-positive) than in epithelial cells (epithelial cell adhesion molecule-positive) in normal lung tissues and LAUD tissues (p < 0.001) (Fig. 1A–D). However, in BM tissue, P4HA2 was more highly expressed in malignant epithelial cells than in fibroblasts (p = 0.002) (Fig. 1E‒F).

**Fig. 2:**
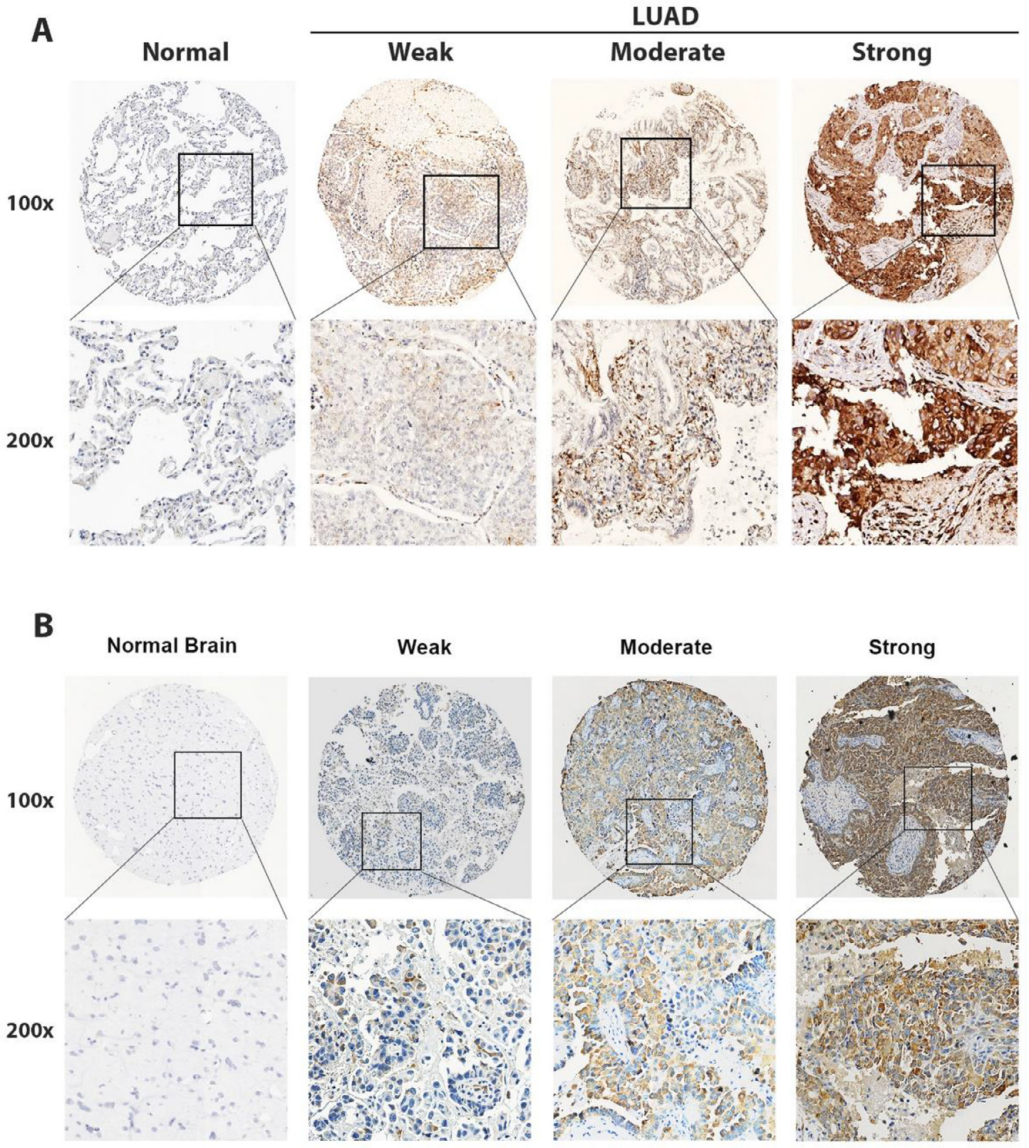
In situ detection of P4HA2 in LUAD and LUAD-BM cases. Representative IHC images of negative, low, moderate, and strong P4HA2 expressions (magnifications, 100 × and 400 ×) in LUAD (A) and LUAD-BM cases (B). P4HA2, Prolyl 4-Hydroxylase subunit Alpha-2; LUAD, Lung Adenocarcinoma; BM, Brain Metastasis; IHC, Immunohistochemistry.

According to IHC results, a strong immune signal for P4HA2 was observed in the cytoplasm of fibroblasts and LUAD tumor cells. The authors rated the staining score of P4HA2 in LUAD-BM cases as weak (< 4) or strong (≥ 4), and 58.4% (66/113) of BM tissues were identified as having high P4HA2 expression (Fig. 2A–B, Fig. S1).

### P4HA2 expression correlated with the clinical characteristics of LUAD

**Fig. 3:**
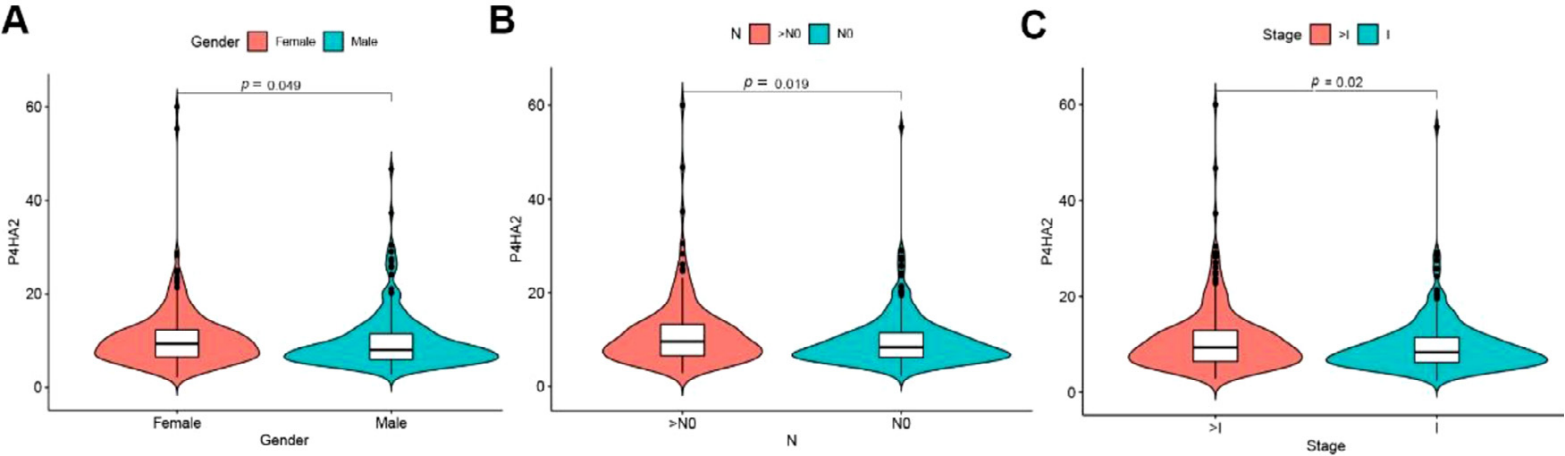
Enrichment analysis of P4HA2 in the TCGA-LUAD dataset. (A) Heatmap of the expression of 859 P4HA2-related genes. (B) Distribution of the Spearman correlation coefficient. Red indicates positive P4HA2-related genes, whereas blue shows negative P4HA2-related genes. (C–D) GO and KEGG analyses of P4HA2 in TCGA-LUAD cases. P4HA2, Prolyl 4-Hydroxylase subunit Alpha-2; LUAD, Lung Adenocarcinoma; TCGA, The Cancer Genome Atlas; GO, Gene Ontology; KEGG, Kyoto Encyclopedia of Genes and Genomes.

The authors examined the correlation between P4HA2 expression levels and clinic-pathological features of patients with TCGA-LUAD. The results showed that P4HA2 messenger (m)-RNA expression was significantly higher in female cases than in male cases with LAUD (p = 0.049). In addition, high P4HA2 mRNA expression occurred frequently in the higher grades of N in the TNM classification (p = 0.019) and higher clinical stage (p = 0.020) (Fig. 3A–C).

### P4HA2 expression predicts poor survival in LUAD and LUAD-BM

**Fig. 4:**
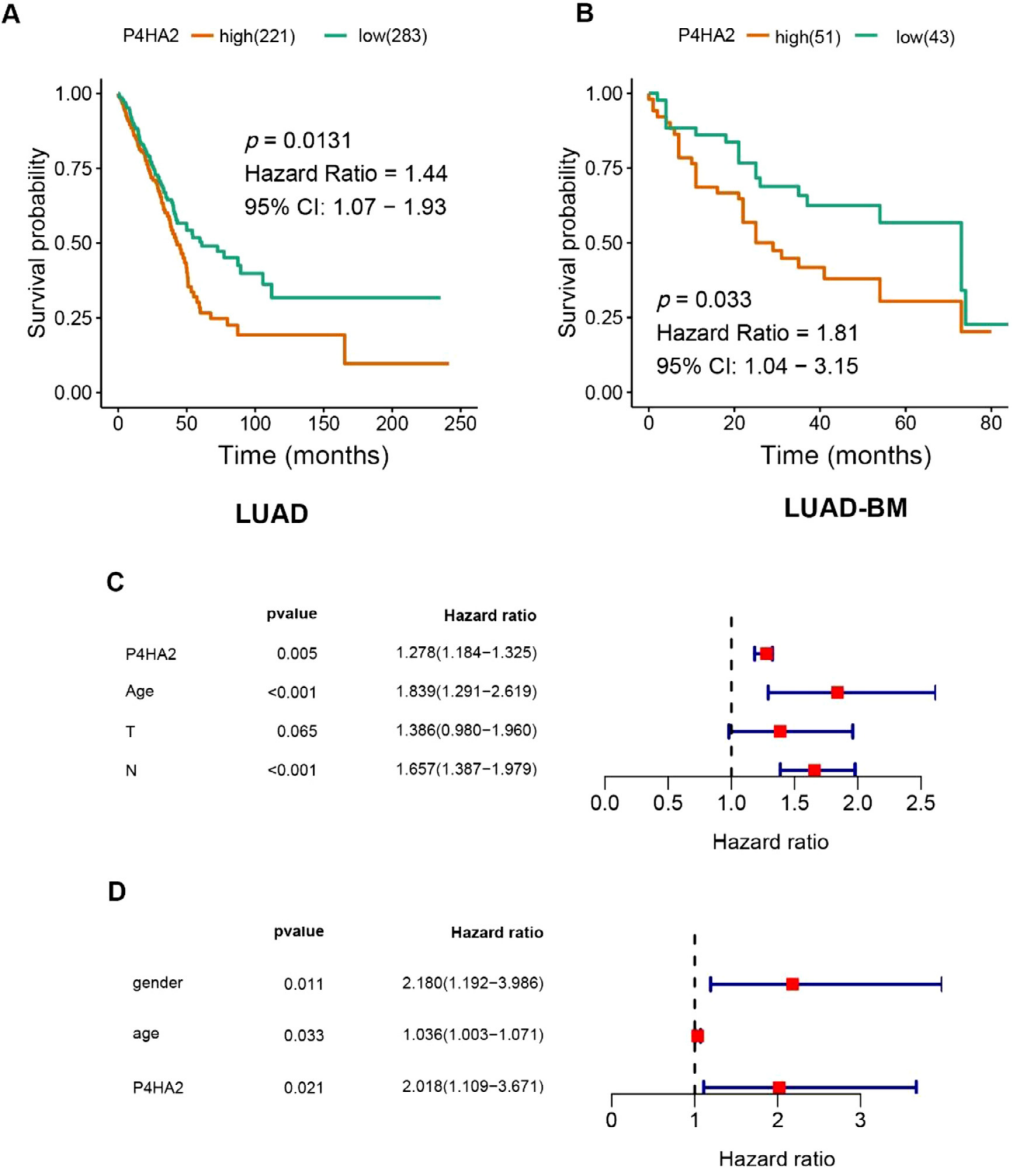
Correlation analysis between P4HA2 expression and clinical characteristics. Differential expression of P4HA2 is significantly related to (A) sex (male patients, n = 237; female patients, n = 277), (B) regional lymph nodes (pN): (N0, n = 331; > N0, n = 171), and (C) clinical stage (stage I, n = 274; > stage I, n = 231). P4HA2, Prolyl 4-Hydroxylase subunit Alpha-2.

Kaplan-Meier survival analysis showed that the expression levels of P4HA2 were negatively related to the prognosis of LUAD (42.9 [95% Confidence Interval ‒95% CI 37.2–50.9] months vs. 61.0 [95% CI 43.1–112.0] months, p = 0.013) (Fig. 4A). Similarly, the results indicated that the high P4HA2 mRNA level showed a strong correlation with the poor prognosis of patients with LUAD-BM (29.0 [95% CI 22.0–37.0] months vs. 73.0 [95% CI 37.0–102.0] months, p = 0.033) (Fig. 4B). Multivariate Cox analysis indicated that high P4HA2 expression was an independent indicator of shorter survival for patients with LUAD and LUAD-BM (p = 0.005 and 0.021, respectively) (Fig. 4C‒D).

### Enrichment analysis of P4HA2 in TCGA-LUAD cases

**Fig. 5:**
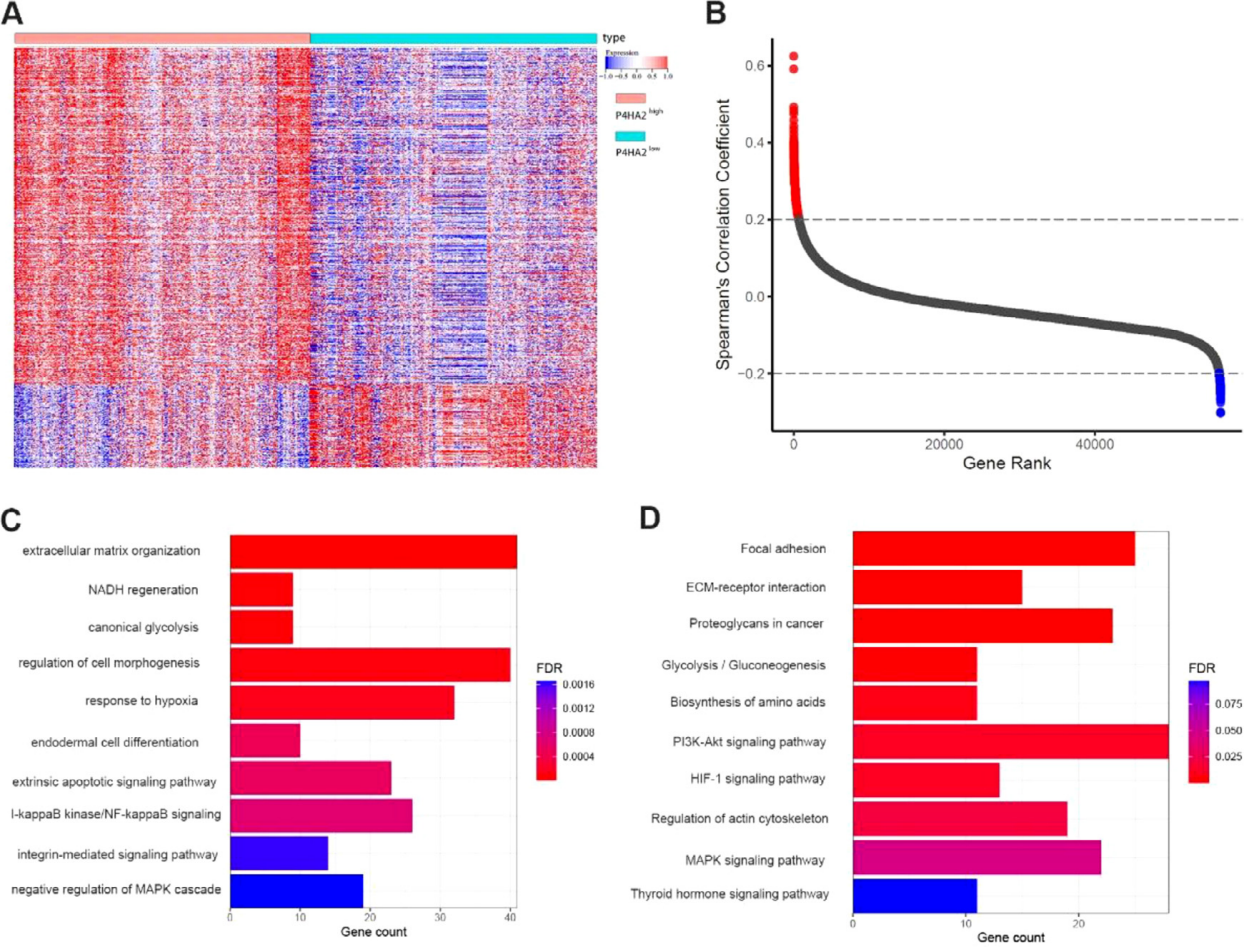
Overexpression of P4HA2 predicts shorter survival for TCGA-LUAD patients (A) and LUAD-BM patients (B**)**. (C–D) Multivariate analysis shows that P4HA2 serves as an independent factor of poor survival in LUAD and LUAD-BM patients. P4HA2, Prolyl 4-Hydroxylase subunit Alpha-2; LUAD, Lung Adenocarcinoma; BM, Brain Metastasis.

In total, 685 and 174 genes were identified as positively and negatively related to P4HA2, respectively (Fig. 5A–B). Based on these significantly P4HA2-related genes, the authors performed Gene Ontology (GO) and Kyoto Encyclopedia of Genes and Genomes (KEGG) analyses. GO analysis showed that P4HA2 was mainly enriched in ECM organization, NADH regeneration, canonical glycolysis, regulation of cell morphogenesis, and response to hypoxia. KEGG pathway analysis showed that P4HA2 was mainly enriched in focal adhesion, ECM receptor interaction, regulation of actin cytoskeleton, cellular metabolism-related pathways, including proteoglycans in cancer, glycolysis/gluconeogenesis, biosynthesis of amino acids, and several important intracellular signaling pathways, e.g., the PI3K-Akt, MAPK, and HIF-1 signaling pathways (Fig. 5C–D).

### Analysis of the immune infiltration and tumor microenvironment

**Fig. 6:**
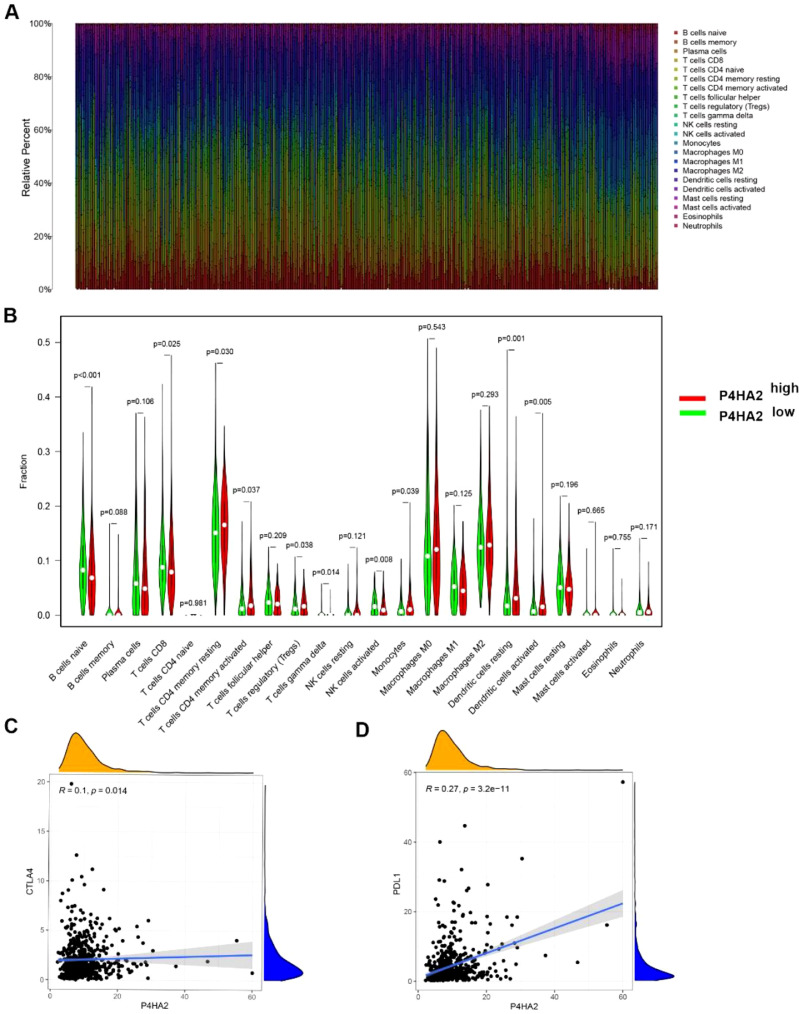
Analysis of P4HA2 in immune-related activities of LUAD. (A) Bar plot showing the proportion of 22 kinds of TIICs in TCGA-LUAD samples. (B) Violin plot showing the ratio differences of 22 TIICs between TCGA-LUAD samples with low or high P4HA2 expression. (C–D) P4HA2 expression is positively correlated with PD-L1 and CTLA4. P4HA2, Prolyl 4-Hydroxylase subunit Alpha-2; LUAD, Lung Adenocarcinoma; TCGA, The Cancer Genome Atlas; TIICs, Tumor-Infiltrating Immune Cells; CTLA4, Cytotoxic T-Lymphocyte-Associated protein 4; PD-L1, Programmed Death-Ligand 1.

Further, the authors analyzed the relationship between P4HA2 expression and tumor-infiltrating immune cells. The comparative content of the immune cell types in each case is presented using a bar plot (Fig. 6A). High levels of P4HA2 were positively correlated with T cells, CD4 memory T-cells, CD4 memory-activated T-cells, regulatory (Tregs) cells, monocytes, resting dendritic cells, and activated dendritic cells (p = 0.029, 0.036, 0.038, 0.039, 0.001, and 0.004, respectively), but negatively correlated with naïve B-cells, CD8+ T-cells, gamma delta T-cells, and activated natural killer cells (p < 0.001, p = 0.002, p = 0.01, and p < 0.001, respectively) (Fig. 6B). Furthermore, to explore the potential of using P4HA2 in immune therapy, the authors examined the relationship between P4HA2, Cytotoxic T-Lymphocyte-Associated protein 4 (CTLA4), and Programmed Death-Ligand 1 (PD-L1) (CD274) and found that CTLA4 and PD-L1 expressions were positively correlated with P4HA2 expression (R = 0.10, p = 0.014; p = 0.27 and p < 0.001, respectively) (Fig. 6C–D).

## Discussion

Although clinical improvement in LAUD treatment has been achieved, cancer metastasis, especially BM and recurrence, remains a challenging clinical problem.^18^ Therefore, exploring novel molecular alterations related to the metastasis of LUAD may provide valuable insights into the design of effective therapeutic strategies. This study showed that P4HA2 is expressed in fibroblasts and epithelial cells, especially in LUAD cells from BMs. Further analysis showed that P4HA2 expression is associated with sex, lymph node metastasis, and tumor grade. Additionally, P4HA2 expression is strongly correlated with patient survival, suggesting that P4HA2 may be considered as a potential biomarker in patients with LUAD, especially for the BM subtype.

P4HA2 is normally expressed in fibroblasts and participates in collagen maturation by hydroxylating the proline residues of procollagen. High P4HA2 expression is associated with poor prognosis in some malignant tumors, such as breast cancer,^10^^,^^19^ cervical cancer,^12^ and prostate cancer.^11^ However, there is a lack of research on the relationship between P4HA2 expression and the prognosis of patients with LUAD, especially for the BM subtype. In the present study, the authors found that P4HA2 was highly expressed in LUAD tumor cells, especially in LUAD BM, based on single-cell analysis and in situ detection. Moreover, P4HA2 expression was positively associated with lymphatic metastasis and a shorter survival time in patients with LUAD. Thus, P4HA2 may serve as a potential biomarker of distant metastasis and poor prognosis in clinical practice.

These results indicated that P4HA2 could promote the metastatic capability of primary LUAD tumor cells. A previous study showed that P4HA2 could accelerate collagen deposition in LUAD, thus creating a stiff ECM,^9^ which was consistent with the result of the GO analysis that P4HA2 was related to ECM organization in the present study. The authors also found that P4HA2 was predominantly involved in several biological functions, e.g., canonical glycolysis, extrinsic apoptotic signaling, NF-kappa B signaling, and response to hypoxia, which are vital biological processes promoting metastasis of tumor cells. Further studies are needed to explore the underlying mechanisms associated with these effects.

Recently, researchers have focused on the Tumor Microenvironment (TME) of lung cancer, which affects disease progression and therapeutic responses.^20^^,^^21^ CTLA4+/PD-1+ Tregs and antigen-presenting cells are associated with immunosuppressive status in the TME.^22^^,^^23^ In addition, CD4+ memory T-cells can accelerate tumor growth by releasing Interleukin (IL)-22 and IL-1β.^24^ Moreover, the accumulation of tumor-associated monocytes has been demonstrated to promote tumor progression, resistance to immune checkpoint therapy, and poor prognosis.^25^ Herein, the authors found that P4HA2 was positively related to these immune cells, suggesting that P4HA2 may be involved in the immune escape of cancer cells. Immune checkpoint inhibitors, such as those targeting programmed cell death protein 1/PD-L1, have improved OS in patients with metastatic Nonsquamous Cell Lung Cancer (NSCLC). In PD-L1-positive individuals, the addition of pembrolizumab increased the 1-year survival rate by 40%–50%.^26^ In addition, ipilimumab (CTLA-4 antibody) and nivolumab (PD-1 antibody) have therapeutic benefits for selected NSCLC (PD-L1 expression ≥ 1% and no EGFR/ALK aberrations).^27^ In conclusion, P4HA2 is positively correlated with PD-L1 and CTLA4. These findings suggest that P4HA2 may serve as a potential marker for immunotherapy in LUAD.

## Conclusion

In the present study, the authors found that P4HA2 is highly expressed in LUAD tumor cells and was a valuable prognostic indicator for LUAD, especially for the BM subtype. It may be involved in the biological activity of distant metastasis of LUAD tumor cells and serve as a potential treatment target.

## Authors' contributions

Q.Y and Y-R.Z designed the study, analyzed the data, and revised the manuscript. X-H.L performed the experiments, analyzed the data, and drafted the manuscript. D.S contributed materials and collected clinical information.

## Ethics approval

This research was approved by the Ethics Committee of the Cancer Hospital, Chinese Academy of Medical Sciences (NCC2021C-516).

## Funding

None.

## Conflicts of interest

The authors declare no conflicts of interest.

## Appendix Supplementary materials

Image, application 1
