# Supplementary material for: High expression of prolyl 4-hydroxylase subunit alpha-2 in lung adenocarcinoma indicates poor prognosis
Source: Clinics (Sao Paulo). 2022 Nov 17;77:100123. doi: 10.1016/j.clinsp.2022.100123 (PMC9678672; doi:10.1016/j.clinsp.2022.100123)

**CLINICS-D-22-00291 – Supplementary Material**

**Figure S1** Representative IHC images of negative, low, moderate, and strong P4HA2 expressions (magnifications 200×) in LUAD tissues.


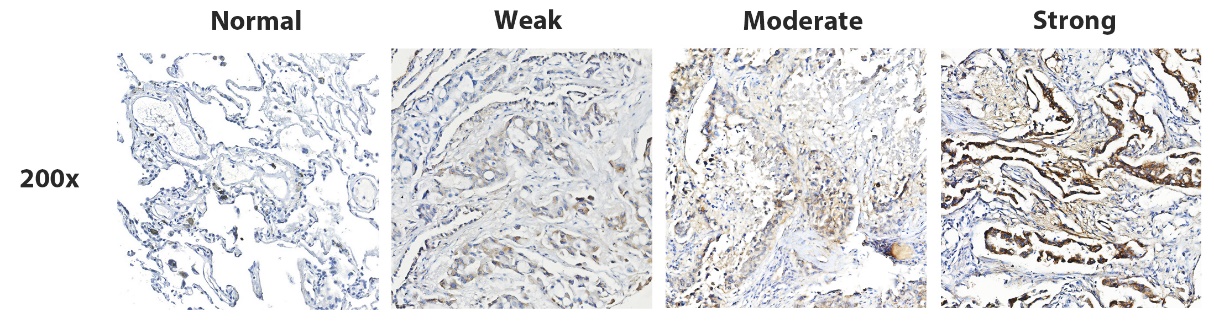

Supplement: Supplementary file 1 [file mmc1.docx]
